# Framework to Detect Schizophrenia in Brain MRI Slices with Mayfly Algorithm-Selected Deep and Handcrafted Features

**DOI:** 10.3390/s23010280

**Published:** 2022-12-27

**Authors:** K. Suresh Manic, Venkatesan Rajinikanth, Ali Saud Al-Bimani, David Taniar, Seifedine Kadry

**Affiliations:** 1 National University of Science and Technology, Muscat P.O. Box 112, Oman; 2 Department of Computer Science and Engineering, Division of Research and Innovation, Saveetha School of Engineering, Saveetha Institute of Medical and Technical Sciences, Chennai 602105, India; 3 Faculty of Information Technology, Monash University, Wellington Rd, Clayton, VIC 3800, Australia; 4 Department of Applied Data Science, Noroff University College, 4612 Kristiansand, Norway; 5 Artificial Intelligence Research Center (AIRC), Ajman University, Ajman P.O. Box 346, United Arab Emirates; 6 Department of Electrical and Computer Engineering, Lebanese American University, Byblos P.O. Box 36, Lebanon

**Keywords:** schizophrenia, brain MRI, VGG19, Markov random field, local binary pattern, disease detection

## Abstract

Brain abnormality causes severe human problems, and thorough screening is necessary to identify the disease. In clinics, bio-image-supported brain abnormality screening is employed mainly because of its investigative accuracy compared with bio-signal (EEG)-based practice. This research aims to develop a reliable disease screening framework for the automatic identification of schizophrenia (SCZ) conditions from brain MRI slices. This scheme consists following phases: (i) MRI slices collection and pre-processing, (ii) implementation of VGG16 to extract deep features (DF), (iii) collection of handcrafted features (HF), (iv) mayfly algorithm-supported optimal feature selection, (v) serial feature concatenation, and (vi) binary classifier execution and validation. The performance of the proposed scheme was independently tested with DF, HF, and concatenated features (DF+HF), and the achieved outcome of this study verifies that the schizophrenia screening accuracy with DF+HF is superior compared with other methods. During this work, 40 patients’ brain MRI images (20 controlled and 20 SCZ class) were considered for the investigation, and the following accuracies were achieved: DF provided >91%, HF obtained >85%, and DF+HF achieved >95%. Therefore, this framework is clinically significant, and in the future, it can be used to inspect actual patients’ brain MRI slices.

## 1. Introduction

The brain is one of the chief organs of humans. The abnormality in the brain causes mild to severe unrecognized problems, and untreated brain abnormality will lead to various other problems [1,2,3]. Therefore, the proposed research considers the schizophrenia (SCZ) diagnosis.

SCZ, a severe mental disorder, typically affects a person’s thinking and behavioral capability. The occurrence rate of this disease listed it in the top 10 illnesses in the global burden of diseases reported by the World Health Organization (WHO) [4]. It is generally diagnosed in men (aged early 20 years) and women (aged 20 to 30 years), and its symptoms are not found in humans less than 12 and more than 40 years old [5].

The various causes which initiate the disorder in teens include genetics (parent or sibling having the illness), environmental condition, troubles with brain chemicals, and usage of mind-altering drugs will increase the occurrence rate of SCZ in humans [6,7]. The recent WHO report confirms that nearly 20 million people globally are suffering from mild to severe disease, and appropriate diagnosis and treatment are necessary to reduce the disease impact [8,9].

Common symptoms in most patients include hallucinations, irregular behavior, speech problems, and emotional instability. If these symptoms are noticed, the patient can undergo a clinical examination to confirm the disease. Therefore, controlling the disease with appropriate treatment procedures is essential. Unfortunately, the WHO report also verifies that around 60% of patients suffering from SCZ are not receiving the appropriate diagnostic facility and treatment in low- and middle-income countries. Because of this, SCZ patients are dying two to three times faster than ordinary people.

Clinical-level assessment of the patient with SCZ is mandatory, and the traditional detection procedures must be modified when significant improvement is achieved. In this case, electroconvulsive therapy and transcranial magnetic stimulation are the two most clinically approved procedures, and electroconvulsive therapy is widely adopted as the gold standard clinical methodology commonly implemented to detect the SCZ. The typical brain signal (EEG) and brain image are collected with a recommended protocol which always provides superior results on the chosen medical data. The EEG-supported SCZ detection is a simple and commonly considered methodology due to its reduced cost and non-invasive nature. However, the information collected from this scheme is complex, and the complexity will increase when a multi-channel EEG is considered to diagnose the disease. Hence, bioimaging (MRI and fMRI) schemes are used during the screening, and it is found that the MRI scheme is efficient in detecting the disease compared with its alternatives. Hence, the proposed work considered artificial intelligence (AI)-based methods, which are used to improve the overall performance during decision making and treatment. The cost of AI-based methods is lower compared with the traditional SCZ screening process.

This research aims to improve a reliable disease diagnostic framework to detect the disease using MRI slices. When the disease is recognized in its early phase, appropriate treatment procedures can be employed to reduce the impact. The developed framework consists of the following phases: (i) collection and pre-processing of the test imagery, (ii) pre-trained deep learning (PDL) scheme execution to mine the deep features (DF), (iii) implementation of a chosen handcrafted feature, (HF) mining technique to get essential features, such as gray level co-occurrence matrix (GLCM) and local binary patterns (LBP), (iv) mayfly optimization algorithm (MOA) supported feature optimization, (v) serial feature concatenation to combine DF and HF, and (vi) classification and validation using binary classifiers with five-fold cross-validation. This framework employs the VGG16 as the prime PDL to improve the disease detection process, and its performance is validated with other pre-trained schemes in the literature.

To extract the HF, this study implemented the following protocol (i) MOA-based Otsu’s thresholding and Markov random field (MRF)-based segmentation to get the gray matter (GM) and white matter (WM) from the brain MRI slice and mining the GLCM from GM and WM images, and (ii) LBP-based image enhancement with mining of various weights (W = 1 to 4) and features. The proposed scheme was experimentally investigated using 40 volunteers’ (20 controlled and 20 SCZ class) images collected from [10,11]. The considered test images were in 3D form. The necessary number of 2D slices was extracted with a chosen technique, and every image was resized to an appropriate dimension. In order to improve the diagnostic accuracy, a threshold filter was employed to remove the skull section from the test image, and a skull-stripped brain MRI slice was then considered for the assessment.

The contributions of this research are as follows:

- Automatic classification of MRI slices into controlled and schizophrenia using chosen binary classifiers.
- Improving the accuracy in MRI slice evaluation using deep- and machine-learning features.
- Mayfly-algorithm-based feature selection to avoid overfitting issue.

This study is presented as follows: Section 2 presents the earlier research information, Section 3 shows the methodology employed, and Section 4 and Section 5 presents the experimental result and conclusion, respectively.

## 2. Related Earlier Works

Early detection of SCZ is necessary to plan and execute the treatment to reduce the impact of the disease on patients. Further, continuous medication and treatment to help the patient recover from the severity of the disease. In the literature, several SCZ detection schemes were proposed to recognize the disease using bio-signal and bio-image-based procedures.

Siuly et al. [12] discussed the detection of SCZ using the EEG signal and achieved improved diagnostic accuracy. Jahmunah et al. [13] proposed detecting the SCZ using non-linear signal processing procedures using multi-channel EEG signals. This was a binary classification to categorize the signals into specific/disease classes. Krishnan et al. [14] discussed the detection of SCZ using the multi-channel EEG processed with intrinsic mode functions (IMF). This work implemented a binary classification to detect the disease with superior accuracy. The recent work of Arunmozhi et al. [15] implemented a joint thresholding and segmentation procedure to extract and evaluate the SCZ from brain MRI slices. Finally, Cetin-Karayumak et al. [16] presented a method to discuss the white matter (WM) abnormality in the brain in SCZ patients. This work presented a detailed examination using diffusion MRI slices.

Along with the EEG, brain MRI-based SCZ diagnosis was also widely discussed by researchers, and these works yielded improved results compared with EEG-based approaches. Oh et al. [17] discussed the detection of SCZ in fMRI slices with the DL scheme. Endres et al. [18] presented a detailed discussion of SCZ detection using EEG and MRI, and this integrated procedure helped achieve an enhanced diagnosis over earlier methods.

Noor et al. [19] presented a detailed review of detecting various brain abnormalities using MRI slices. This work also presented the existing procedures to detect the SCZ with improved accuracy and confirmed the need for the diagnosis’s artificial intelligence (AI) technique.

All the above discussed a chosen AI technique to detect the SCZ using EEG and MRI slices. This work confirms the need for a reliable SCZ detection procedure to reduce the disease diagnostic burden in hospitals, particularly in low- and middle-income countries.

The proposed research aims to develop a novel SCZ detection scheme by integrating the DF and HF to achieve better accuracy. The developed framework helps to detect the SCZ using the MRI slices. The report achieved with this scheme is considered an initial report and must be verified and confirmed medical professionals. The information achieved from the system can support doctors during the treatment planning and implementation.

## 3. Methodology

**Figure 1:**
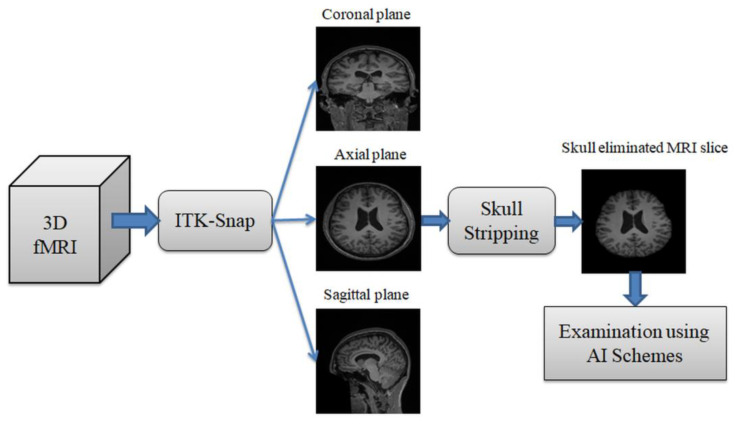
Image processing technique employed to get necessary 2D slices.

This section of the work presents the methodology employed to detect the SCZ using the MRI slices. The proposed work was implemented on the 2D slices extracted from the considered SCZ database. The dataset was in 3D form and 3D to 2D conversion was implemented using the ITK-Snap software [20,21]. This conversion helps to separate the 3D MRI into an axial, coronal, and sagittal plane, and the axial plane was examined in this study for the assessment. This work also employed an artifact removal procedure to eliminate the skull section, as depicted in Figure 1 [22].

### 3.1. Proposed Scheme

**Figure 2:**
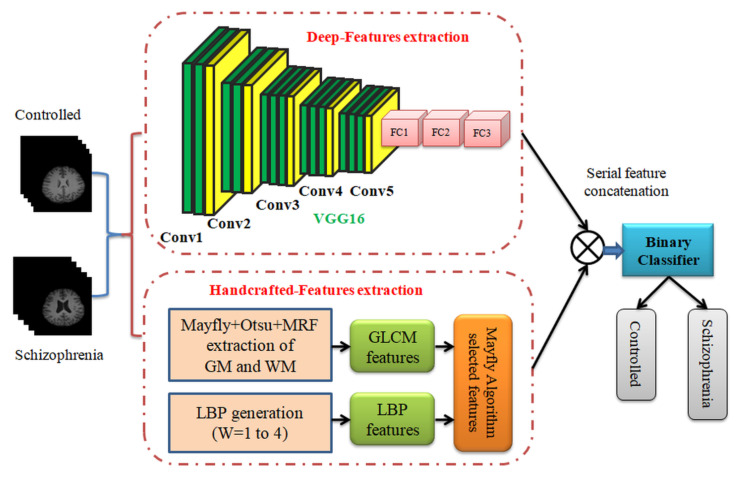
Proposed SCZ detection procedure.

Figure 2 presents the SCZ examination technique implemented in this research. Initially, the pre-processed test images were examined and necessary DF and HF were extracted. To extract the DF, the PDL scheme (VGG16) was employed. This scheme initially supplied a 1D feature vector of dimension 1×1×4096, and after the necessary dropout (50%), this feature was then reduced to a value of 1×1×1024 features. These features were then considered to train and validate the classifiers to detect the SCZ. This framework also consisted of an HF extraction procedure to get the GLCM and LBP features, and optimal values of these features were then identified using MOA. The selected HF were then serially combined with the DF with a dropout rate of 50% (i.e., 1×1×512 features), and the concatenated features (DF+HF) were then considered to validate the SCZ detection process using the binary classifiers employed with 5-fold cross-validation. The implemented framework confirms that the classifier accuracy achieved using DF+HF is better (>95%) compared with that of other procedures suggested in this research work.

### 3.2. Schizophrenia Database

**Figure 3:**
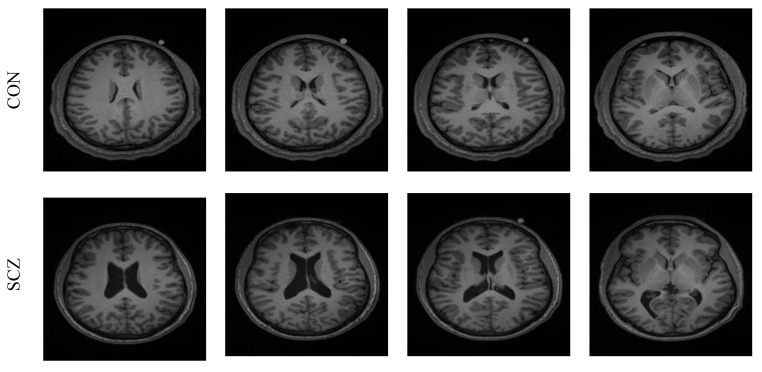
Sample test images of CON and SCZ class.

The performance of the developed system was tested and validated using the clinical grade SCZ MRI dataset from [10,11]. This dataset consists of 99 volunteers’ 3D images with the following categories: SCZ, controlled (CON), SCZ-sibling, and CON-sibling. These images were collected from male and female volunteers whose racial demographics included White and African American. The earlier works on this dataset can be found in [21]. In this work, 20 3D MRI images were considered from the CON/SCZ class, and from every volunteer’s 3D data, 30 slices (axial plane) were extracted using ITK-Snap, and every image was then resized to a dimension of 224×224×3 pixels. The skull section in these images was then eliminated using the thresholding filter/skull stripping algorithm discussed in [22,23]. The test images considered in this work are presented in Table 1 and the sample images are presented in Figure 3.

### 3.3. Deep Feature Extraction

In the literature, a considerable number of earlier works are available to provide the necessary information about the PDL schemes employed to examine a variety of medical images (gray/RGB scale) [24,25]. The image examination procedures proposed with VGG16 confirm its merits, such as simple architecture, easy training and validation, and better accuracy compared with other PDL schemes. Hence, in this work, the pre-trained VGG16 was adopted to extract the DF from the brain MRI slices, and during this task, the following initial parameters were assigned: conventional augmentation to boost the number of test images, learning rate with a value of 1 × 10^−5^ to obtain better accuracy, linear dropout rate (LDR) during training with an Adam optimizer. The number of iterations was chosen as 4000 and the total epochs were fixed as 50. In the fully connected (FC) layers, a 50% dropout rate was assigned, which gives a DF dimension of 1×1×1024, and these features were considered to train and validate the disease detection performance of VGG16. During this process, a 5-fold cross-validation was assigned and the best value among the trials was chosen as the final result of the PDL scheme. A similar procedure was then repeated with other PDL, such as VGG19. AlexNet, ResNet18, ResNet50, ResNet101, and Inception-V3 were used in this study [26]. Initially, SoftMax was considered for the image classification and a similar procedure was then repeated using other binary classifiers.

### 3.4. Handcrafted Feature Extraction

HF plays a major role in machine-learning schemes and the extracted features were considered to support the automated detection of diseases from medical images. In this work, the necessary HF, such as GLCM and LBP, were extracted. 

#### 3.4.1. Gray Level Co-Occurrence Matrix

In the literature, the GLCM features are widely considered to detect the disease using medical images [27,28,29,30]. In this work, the GLCM features were extracted from the gray matter (GM) and white matter (WM) sections of the brain MRI. This extraction was performed using the joint thresholding and segmentation implemented with MOA, Otsu, and MRF. The earlier works with a similar technique can be found in [22,23].

**Figure 4:**
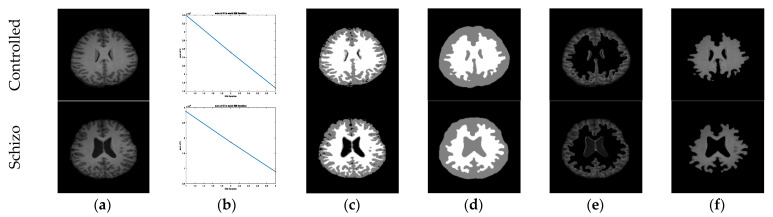
Separation of brain MRI slice into GM and WM: (**a**) Image; (**b**) Convergence; (**c**) Initial; (**d**) Final; (**e**) GM; (**f**) WM.

The proposed work implemented the MOA- and Otsu-based tri-level thresholding to enhance the image and MRF-based pixel improvement and segmentation technique. The various stages involved in this process are as follows: (i) implementation of MOA and Otsu’s thresholding to pre-process the image, (ii) MRF-based image enhancement and pixel-based separation, (iii) obtaining the GM and WM sections, and (iv) GLCM separately feature extraction from GM and WM images. This is an automated scheme and helps to separate the brain MRI slice into two sections. The attained result with the proposed scheme can be found in Figure 4. Figure 4a depicts the sample test image and Figure 4b depicts the reduced energy function during the MRF process, Figure 4c,d depicts the initial and final enhance image labels, and Figure 4e,f presents the extracted GM and WM sections, respectively.

#### 3.4.2. Local Binary Pattern

LBP is one of the commonly employed quality improvement practices, and in this work, the weighted LBP proposed by Gudigar et al. [31] was employed. The LBP is a simple and capable technique to enhance the textural components of the image. The necessary LBP was formed by relating the inmost pixel with neighbor pixels.

In LBP, the average local gray level can be calculated as:

(1) $$ALGL=\frac{\sum_{i=1}^{8}\left(N_{gi}+C_{g}\right)}{9}$$

where $C_{g}$ is the gray level of the midpoint pixel, $N_{gi}$ is the grey level of neighbor pixels, and *i* = 1, 2, …, 8.

For a typical image, the global weighted gray level can be computed with:

(2) global weighted graylevel =β(μ+σ)

where μ denotes the mean, σ represents the standard deviation, and β is a control variable with values, such as 1, 2, 3, …

The weighted LBP for an image can be computed as:

(3) $$LBP=\sum_{q=0}^{Q-1}s(N_{gq}-C_{g})2^{q}$$

where $N_{gq}$ shows the neighboring gray values, Q is the number of neighbors, q=0,1,2,…,Q−1, and s is 0 or 1 based on the magnitude and threshold.

Other essential information on the LBP can be found in [32,33,34].

**Figure 5:**
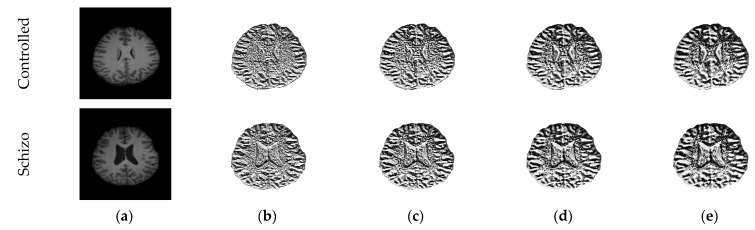
Sample LBP images for W = 1 to 4: (**a**) Image; (**b**) W = 1; (**c**) W = 2; (**d**) W = 3; (**e**) W = 4.

The LBP pattern achieved for the sample test image can be found in Figure 5. In this work, the LBP weight (W) was chosen with a value of 1 to 4, and an enhanced image was then considered to extract a feature with dimension 1×1×59 features.

### 3.5. Mayfly Algorithm Selected Features

MOA is one of the recently proposed soft computing techniques developed by integrating the best factions of the firefly algorithm (FA), particle swarm optimization (PSO), and genetic algorithm (GA), the mathematical expression for this algorithm is discussed below:

Assuming that MOA has equal male (M) and female (F) flies, which are randomly distributed in a D-dimensional search location, every fly is symbolized by i=1,2,…,n (forn = 30). During the exploration stage, each fly is permitted to join at the finest location ($G_{best}$). Afterward, M is approved to meet at $G_{best}$ by altering its location and speed. The junction M close to the finest place will be decided by the Cartesian distance (CD) enlarged with respect to iteration. This process is shown in Equations (4) and (5):

(4) $$P_{i}^{t+1}=P_{i}^{t}+V_{i}^{t+1}$$

(5) $$V_{i,j}^{t+1}=V_{i,j}^{t}+C_{1}*e^{-\beta D_{p}^{2}}(p_{best_{i,j}}-P_{i,j}^{t})+C_{2}*e^{-\beta D_{g}^{2}}(G_{best_{i,j}}-P_{i,j}^{t})$$

where $P_{i}^{t}$ and $P_{i}^{t+1}$ are initial and final locations, $V_{i}^{t+1}$ and $V_{i,j}^{t+1}$ initial and final velocities, respectively.$C_{1}=1$ and $C_{2}=1.5$ indicate local and global learning parameters. β=2, $D_{p}$, and $D_{g}$ are the CD. When the update in flies persists, every M will attain $G_{best}$ and performs a velocity update to attract F by performing a unique nuptial dance.

The velocity update during this process can be defined as:

(6) $$V_{i,j}^{t+1}=V_{i,j}^{t}+d*R$$

where nuptial dance (*d*) = 5 and R = random numeral [−1,1].

When the search by M is over, each F is allowed to find a M converged at $G_{best}$.

The expression for position and velocity update for the F is depicted below.

(7) $$P^{\prime }{}_{i}^{t+1}=P^{\prime }{}_{i}^{t}+V^{\prime }{}_{i}^{t+1}$$

(8) $$F^{\prime }{}_{i,j}^{t+1}=\left\{\begin{array}{l} F^{\prime }{}_{i,j}^{t}+C_{2}e^{-\beta D_{mf}{}^{2}}(M_{i,j}^{t}-Y_{i,j}^{t}){\;\;\;\;\;\;\mathrm{if}\;O(F}_{i})>{O(M}_{i})\;\\ F^{\prime }{}_{i,j}^{t}+W*r{\;\mathrm{if}\;O(F}_{i})\leq {O(M}_{i})\; \end{array}\right.\;$$

where O = maximized objective value.

When the iteration improves, every F will reach the M and the offspring generation takes place. Other information on MOA can be found in [35,36,37].

**Figure 6:**
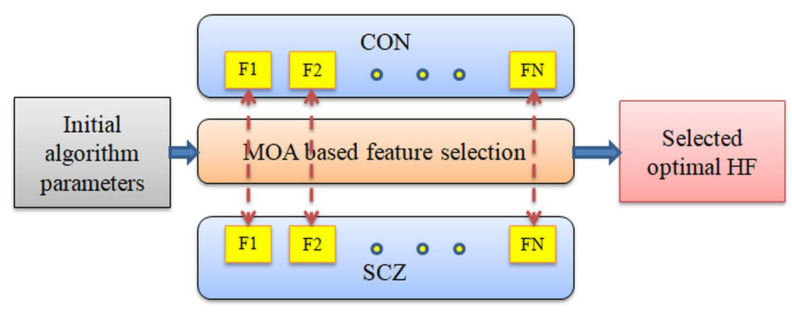
Selection of optimal HF using MOA.

The objective of this study is to select the best features based on the CD of the CON and SCZ images. This process is shown in Figure 6.

The MOA parameters were assigned as follows, the number of flies = 30, total iterations = 3000, objective value = maximization of CD, and terminating criteria = maximum iteration. Other parameters were assigned as in [38].

In this work, the MOA is considered to select the finest HF by comparing the features of CON and SCZ class images, and this process is presented in Equations (9)–(15)

(9) $$GLCM_{WM_{\left(1\times 1\times 25\right)}}=GLCM_{WM1},GLCM_{WM2,}\ldots ,GLCM_{WM25}$$

(10) $$GLCM_{GM_{\left(1\times 1\times 25\right)}}=GLCM_{GM1},GLCM_{GM2,}\ldots ,GLCM_{GM25}$$

(11) $$LBP_{W1_{\left(1\times 1\times 59\right)}}=LBP_{W1_{1}},LBP_{W1_{2}}\ldots ,LBP_{W1_{59}}$$

(12) $$LBP_{W2_{\left(1\times 1\times 59\right)}}=LBP_{W2_{1}},LBP_{W2_{2}}\ldots ,LBP_{W2_{59}}$$

(13) $$LBP_{W3_{\left(1\times 1\times 59\right)}}=LBP_{W3_{1}},LBP_{W3_{2}}\ldots ,LBP_{W3_{59}}$$

(14) $$LBP_{W4_{\left(1\times 1\times 59\right)}}=LBP_{W4_{1}},LBP_{W4_{2}}\ldots ,LBP_{W4_{59}}$$

(15) $$HF_{\left(1\times 1\times 286\right)}=GLCM_{WM}+GLCM_{GM}+LBP_{W1}+LBP_{W2}+LBP_{W3}+LBP_{W4}$$

In this work, the MOA-based feature selection was adopted to select 1×1×103 HF from 1×1×286 features.

### 3.6. Serial Features Concatenation

Serial feature concatenation is one of the commonly adopted features uniting the procedures, which is employed to combine the HF and DF. In this work, the DF of VFF16 was initially reduced to 1×1×512 by implementing a feature ranking and a 50% dropout process. The reduced feature was then combined with the optimal HF of value 1×1×103 to get the concatenated feature shown in Equation (16). These features were then considered to train and validate the considered disease detection scheme [39,40,41,42,43].

(16) $$Concatinated\;features{\;}_{\left(DF+HF\right)=\left(1\times 1\times 512\right)+\left(1\times 1\times 103\right)=1\times 1\times 615}$$

### 3.7. Classification and Validation

The performance of the disease detection system depends on the scientific measures computed using an experimental investigation. The performance of the proposed scheme was confirmed using an experimental investigation, and during this investigation, the binary classifiers, such as SoftMax, decision tree (DT), logistic regression, Naïve Bayes, SVM linear kernel, boosted trees and K-nearest neighbour (KNN) were considered. During this investigation, the necessary measures, such as the true positive (TP), false negative (FN), true negative (TN), and false positive (FP) were initially computed and from these values, other values, such as accuracy (ACC), precision (PRE), sensitivity (SEN), specificity (SPE), negative predictive value (NPV), and F1-Score (FS) were achieved. The expression for these values can be found in Equations (17)–(22) [39,40]:

(17) $$ACC=\frac{TP+TN}{TP+TN+FP+FN}$$

(18) $$PRE=\frac{TP}{TP+FP}$$

(19) $$SEN=\frac{TP}{TP+FN}$$

(20) $$SPE=\frac{TN}{TN+FP}$$

(21) $$NPV=\frac{TN}{TN+FN}$$

(22) $$FS=\frac{2TP}{2TP+FN+FP}$$

## 4. Result and Discussion

This section of the paper demonstrates the results attained using an Intel i7 2.9 GHz processor with 12GB RAM and 4GB VRAM equipped with MATLAB^®^.

**Figure 7:**
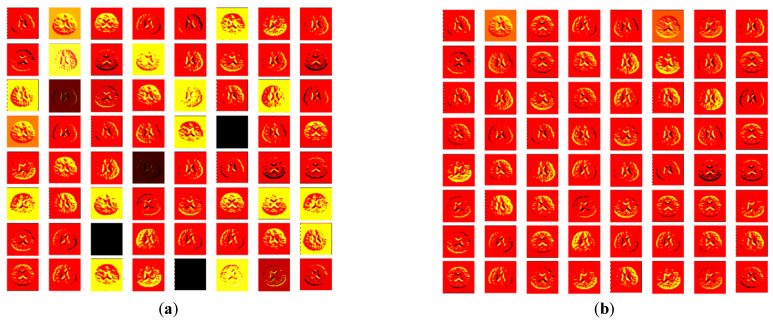
Initial convolution layer result of VGG16: (**a**) Convolution (8×8=64); (**b**) MaxPool (8×8=64 ).

In this proposed work, the considered system was tested and its performance was confirmed using the images presented in Table 1. Initially, the performance of the VGG16 was verified using considered images. During this process, the 2D MRI slices with dimension 224×224×3 pixels were considered and 420 images along with specified augmentation (rotation of images with an angle of $\pm 60^{o}$ in steps of $10^{o}$) were initially performed to train the PDL scheme. After the training, its disease detection performance was then verified using the SoftMax classifier with a 5-fold cross-validation. Figure 7 presents the sample results extracted from the initial convolution layer of VGG16. Figure 7a,b presents the convolutional and MaxPool layer values, which are transferred to the next level of the PDL, this process continues unti the FC layer offers a feature vector with a dimension 1×1×1024. Finally, the SoftMax layer considers these features to categorize the testing images into CON/SCZ classes.

**Figure 8:**
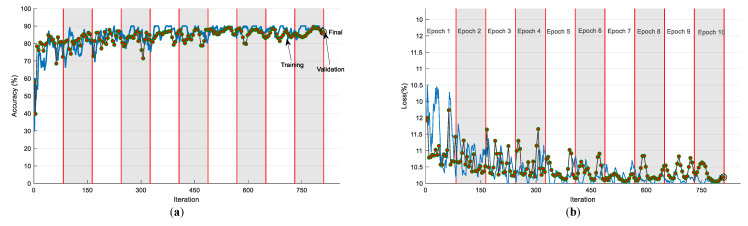
Convergence of the training and validation operation for a trial with VGG16: (**a**) Accuracy; (**b**) Loss.

**Figure 9:**
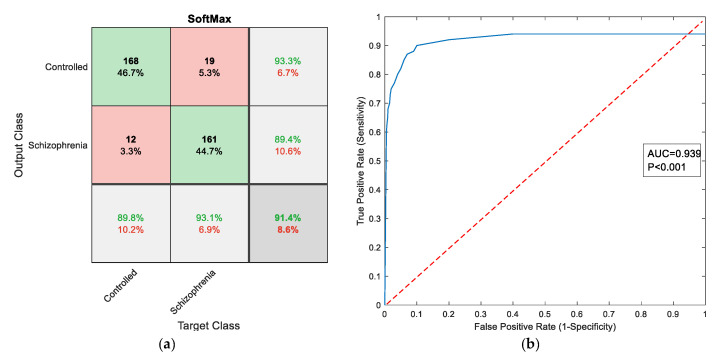
Sample results attained with VGG16: (**a**) Confusion matrix; (**b**) ROC curve.

Figure 8 depicts the convergence of the VGG16’s training and validation operation. From Figure 8a, it can be noted that the accuracy is around 90% and the loss value is closer to 10%, as in Figure 8b. This process was repeated five times and the best value achieved during this process (trial 4 value) was considered the final result. The sample confusion matrix and ROC curves for this process are depicted in Figure 9a,b, respectively.

**Figure 10:**
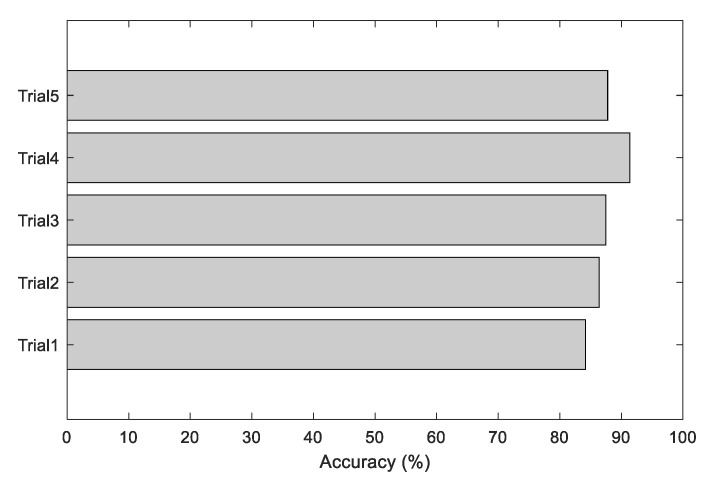
Accuracy values achieved during various trials.

**Figure 11:**
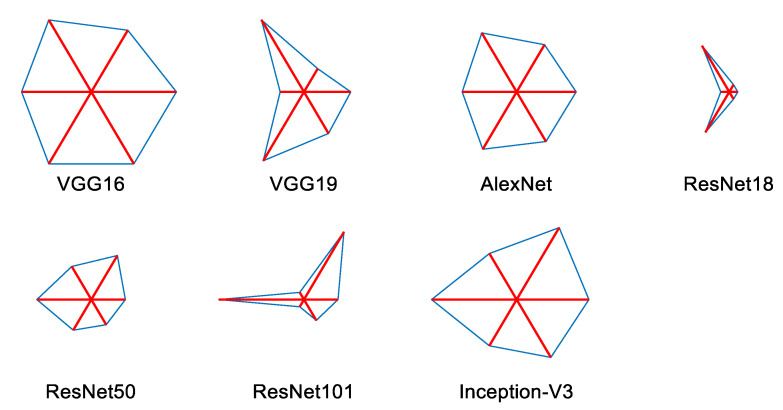
Glyph plot to verify the overall performance of various schemes.

Various performance values achieved during the 5-fold cross-validation are presented in Table 2, and the corresponding accuracy is depicted in Figure 10. From this Table and Figure, it is confirmed that the performance of trial 4 is better for VGG16, and this value was chosen as the final output. A similar procedure was repeated with other existing pre-trained DL schemes in the literature, and the results achieved for a SoftMax classifier are depicted in Table 3. The overall performance of the PDL schemes with the SoftMax classifier is presented as a glyph plot in Figure 11. This information also confirms that the disease detection accuracy of VGG16 is better compared with other PDL methods. In Figure 11, the image with a broader area is considered the best result, which confirms that VGG16 offers better overall results on the considered brain MRI database.

After verifying the disease detection performance of the PDL scheme, the binary classification was once again implemented using the optimally selected HF and its outcome was then verified. During this operation, 1×1×103 features were considered to verify disease detection with various binary classifiers. Table 4 presents the results achieved with various classifiers considered in this research work. For DT and KNN, its variants, such as coarse, medium, and fine were considered, and the results depicted in this table confirm that the optimal HF feature helped to achieve a classification accuracy of up to 85.2778% (DT-fine) and this value is lower compared with the VGG16 with SoftMax classifier.

In order to improve the accuracy achieved using individual DF and HF, a commonly adopted serial concatenation was then employed (DF+HF) and the disease detection process was once again repeated with various binary classifiers. During this process, the feature sub-set with a dimension of 1×1×615 features was then considered and the image classification task was once again repeated. 

**Figure 12:**
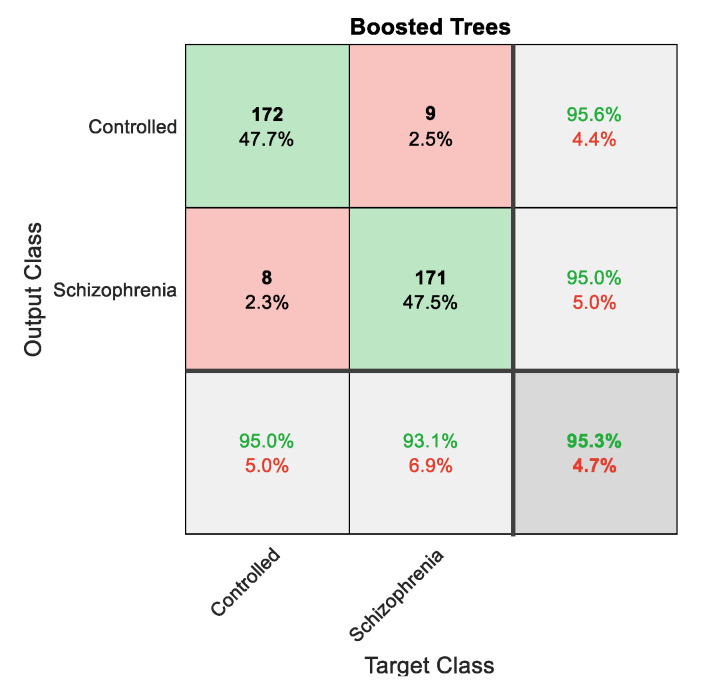
Confusion matrix achieved for boosted trees classifier.

**Figure 13:**
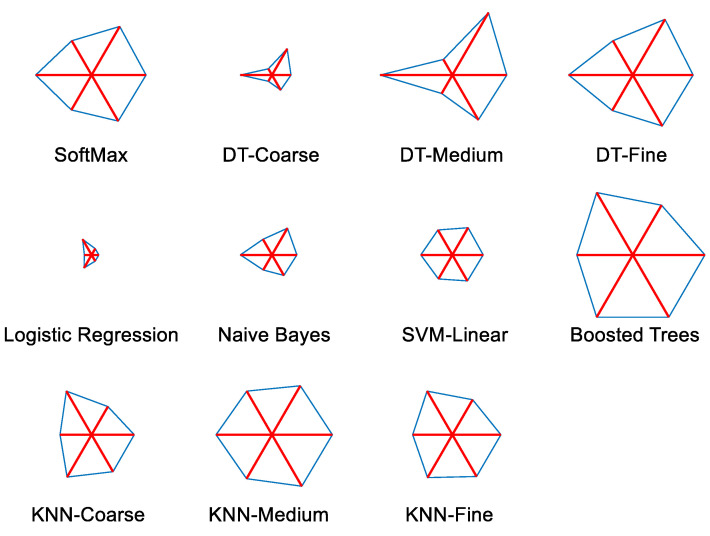
Glyph plot constructed using the overall performance achieved during DF+HF based classification.

The classification results achieved with concatenated features (DF+HF) are depicted in Table 5, and these results confirm that the overall result by boosted trees is better (accuracy > 95%) compared with other methods. The confusion matrix achieved for this classifier is depicted in Figure 12. Figure 13 presents the glyph plot constructed using Table 4 values. This also confirms that the boosted trees classifier outperforms other classifiers considered in this research work.

In the proposed research, a novel procedure was developed to classify the brain MRI slices into CON and SCZ classes, and this procedure helped to achieve a classification accuracy of >95%. In the future, the proposed scheme can be improved by considering other handcrafted features existing in the literature. Further, the performance of the proposed scheme can be tested and validated on other brain abnormalities, such as brain tumors and ischemic strokes recorded with MRI imaging modalities.

## 5. Conclusions

In recent years, the incidence rate of schizophrenia (SCZ) has increased among teenagers due to various causes, and early diagnosis and treatment is necessary to reduce the impact of this abnormality. Medical image-supported SCZ detection is essential for the appropriate treatment planning and for helping patients to have a better life. The proposed research aims to develop a disease detection system to identify the SCZ class brain MRI slice with better accuracy. The performance of the proposed system was individually tested using (i) DF alone, (ii) HF alone, and (iii) serially concatenated features (DF+HF). The proposed scheme employs the VGG16 architecture to get the necessary DF from the MRI slices, and then the necessary HF is obtained using the MRF segmented images (GM and WM) and LBP patterns. This work also employed the MOA-based optimal feature selection process to reduce the dimension of the HF. The concatenated features with a dimension of 1×1×615 helped to achieve a classification accuracy of >95% with a binary classification executed with the boosted trees classifier. The result achieved with this classifier is better compared with other binary classifiers considered in this research. In the future, the classification result of this scheme can be improved by considering other HF existing in the literature.

## Figures and Tables

**Table 1:** Test images considered in this research work.

Image Class	Image Dimension	Number of MRI Slices Considered		
		Total	Training	Validation
**Controlled**	224×224×3	600	420	180
Schizo	224×224×3	600	420	180

**Table 2:** Performance values achieved with SoftMax during 5-fold cross-validation.

Folds	TP	FN	TN	FP	ACC	PRE	SEN	SPE	NPV	FS
Trial1	155	25	148	32	84.1667	82.8877	86.1111	82.2222	85.5491	84.4687
Trial2	159	21	152	28	86.3889	85.0267	88.3333	84.4444	87.8613	86.6485
Trial3	154	26	161	19	87.5000	89.0173	85.5556	89.4444	86.0963	87.2521
Trial4	168	12	161	19	91.3889	89.8396	93.3333	89.4444	93.0636	91.5531
Trial5	156	24	160	20	87.7778	88.6364	86.6667	88.8889	86.9565	87.6404

**Table 3:** Performance values achieved with other pre-trained schemes with SoftMax classifier.

Deep-Learning Scheme	ACC	PRE	SEN	SPE	NPV	FS
VGG16	91.3889	89.8396	93.3333	89.4444	93.0636	91.5531
VGG19	90.5556	88.4211	93.3333	87.7778	92.9412	90.8108
AlexNet	90.8333	89.3048	92.7778	88.8889	92.4855	91.0082
ResNet18	89.7222	87.8307	92.2222	87.2222	91.8129	89.9729
ResNet50	90.2778	89.1892	91.6667	88.8889	91.4286	90.4110
ResNet101	90.2778	90.0552	90.5556	90.0000	90.5028	90.3047
Inception-V3	91.1111	90.2174	92.2222	90.0000	92.0455	91.2088

**Table 4:** Disease detection performance of binary classifiers with optimal HF.

Binary Classifiers	ACC	PRE	SEN	SPE	NPV	FS
DT-coarse	84.1667	84.3575	83.8889	84.4444	83.9779	84.1226
DT-medium	84.4444	84.0659	85.0000	83.8889	84.8315	84.5304
DT-fine	85.2778	85.0829	85.5556	85.0000	85.4749	85.3186
Logistic regression	83.6111	83.7989	83.3333	83.8889	83.4254	83.5655
Naive Bayes	82.7778	83.1461	82.2222	83.3333	82.4176	82.6816
SVM-linear	83.6111	83.7989	83.3333	83.8889	83.4254	83.5655
Boosted trees	84.7222	83.7838	86.1111	83.3333	85.7143	84.9315
KNN-coarse	83.3333	82.9670	83.8889	82.7778	83.7079	83.4254
KNN-medium	83.0556	82.5137	83.8889	82.2222	83.6158	83.1956
KNN-fine	84.1667	83.9779	84.4444	83.8889	84.3575	84.2105

**Table 5:** Disease detection performance of binary classifiers with DF+HF.

Binary Classifiers	ACC	PRE	SEN	SPE	NPV	FS
SoftMax	94.4444	94.9438	93.8889	95.0000	93.9560	94.4134
DT-coarse	92.7778	93.2584	92.2222	93.3333	92.3077	92.7374
DT-medium	94.4444	95.9770	92.7778	96.1111	93.0108	94.3503
DT-fine	94.7222	95.4802	93.8889	95.5556	93.9891	94.6779
Logistic regression	92.2222	91.7582	92.7778	91.6667	92.6966	92.2652
Naive Bayes	93.0556	93.2961	92.7778	93.3333	92.8177	93.0362
SVM-linear	93.3333	93.3333	93.3333	93.3333	93.3333	93.3333
Boosted trees	95.2778	95.0276	95.5556	95.0000	95.5307	95.2909
KNN-coarse	93.8889	93.4066	94.4444	93.3333	94.3820	93.9227
KNN-medium	94.7222	94.9721	94.4444	95.0000	94.4751	94.7075
KNN-fine	94.1667	93.9227	94.4444	93.8889	94.4134	94.1828

## Data Availability

The brain MRI images considered in this research work can be accessed from https://openneuro.org/datasets/ds000115/versions/00001 (accessed on 15 September 2022).
